# Microglial- neuronal crosstalk in chronic viral infection through mTOR, SPP1/OPN and inflammasome pathway signaling

**DOI:** 10.3389/fimmu.2024.1368465

**Published:** 2024-04-05

**Authors:** Catalina Argandona Lopez, Amanda M. Brown

**Affiliations:** ^1^ Division of Neuroimmunology, Department of Neurology, Johns Hopkins University School of Medicine, Baltimore, MD, United States; ^2^ Division of Neuroimmunology, Department of Neurology and Neuroscience, Johns Hopkins University School of Medicine, Baltimore, MD, United States

**Keywords:** neuroimmunology, neuroinflammation, microglia, latency, human immunodeficiency virus, neurodegeneration, neurological disorders, integrins

## Abstract

HIV-infection of microglia and macrophages (MMs) induces neuronal injury and chronic release of inflammatory stimuli through direct and indirect molecular pathways. A large percentage of people with HIV-associated neurologic and psychiatric co-morbidities have high levels of circulating inflammatory molecules. Microglia, given their susceptibility to HIV infection and long-lived nature, are reservoirs for persistent infection. MMs and neurons possess the molecular machinery to detect pathogen nucleic acids and proteins to activate innate immune signals. Full activation of inflammasome assembly and expression of IL-1β requires a priming event and a second signal. Many studies have demonstrated that HIV infection alone can activate inflammasome activity. Interestingly, secreted phosphoprotein-1 (*SPP1*/OPN) expression is highly upregulated in the CNS of people infected with HIV and neurologic dysfunction. Interestingly, all evidence thus far suggests a protective function of *SPP1* signaling through mammalian target of rapamycin (mTORC1/2) pathway function to counter HIV-neuronal injury. Moreover, HIV-infected mice knocked down for *SPP1* show by neuroimaging, increased neuroinflammation compared to controls. This suggests that *SPP1* uses unique regulatory mechanisms to control the level of inflammatory signaling. In this mini review, we discuss the known and yet-to-be discovered biological links between *SPP1*-mediated stimulation of mTOR and inflammasome activity. Additional new mechanistic insights from studies in relevant experimental models will provide a greater understanding of crosstalk between microglia and neurons in the regulation of CNS homeostasis.

## Introduction

Neurologic and gait disturbances were hallmark features of HIV-1 disease in the 1980s demonstrating the profound negative impact of the virus on central nervous system (CNS) functioning (1). The clinical manifestations of NeuroHIV can include cognitive impairment, depression, anxiety, and deficits in fine motor movements (2, 3). Comprehensive neuropsychological testing is used to identify people with HIV-associated neurocognitive disorder, now more generally known as NeuroHIV, to reflect the changing clinical spectrum of neurologic and psychiatric co-morbidities (4–6). Seminal neuropathology studies on HIV-infected post-mortem human brain tissue identified brain microglia and macrophages (MMs) as the predominant cellular targets of the virus (7–10). Through different mechanisms, HIV-infected monocytes, T-cells, and viral particles cross the blood-brain-barrier, which itself becomes impaired (11–14). Targeted antiretroviral therapies (ART), first introduced in 1996, were highly effective at blocking virus replication and sparing CD4+ T-cell death and immune system dysfunction (15). Many ART regimens reach pharmacological levels in the CSF; however, whether inhibitory concentrations reach regions in the brain parenchyma, where HIV-infected MMs reside, remains unclear (16–18). Additionally, yolk sac-derived microglia are relatively long-lived cells with a turnover of many months, and their capacity for self-renewal provides a sanctuary for HIV in brain tissue (19–21). Even under conditions of low-level HIV gene expression, immune activation in the form of increased circulating pro-inflammatory cytokines and immune markers are present in people with HIV on ART (22, 23).

**Figure 1:**
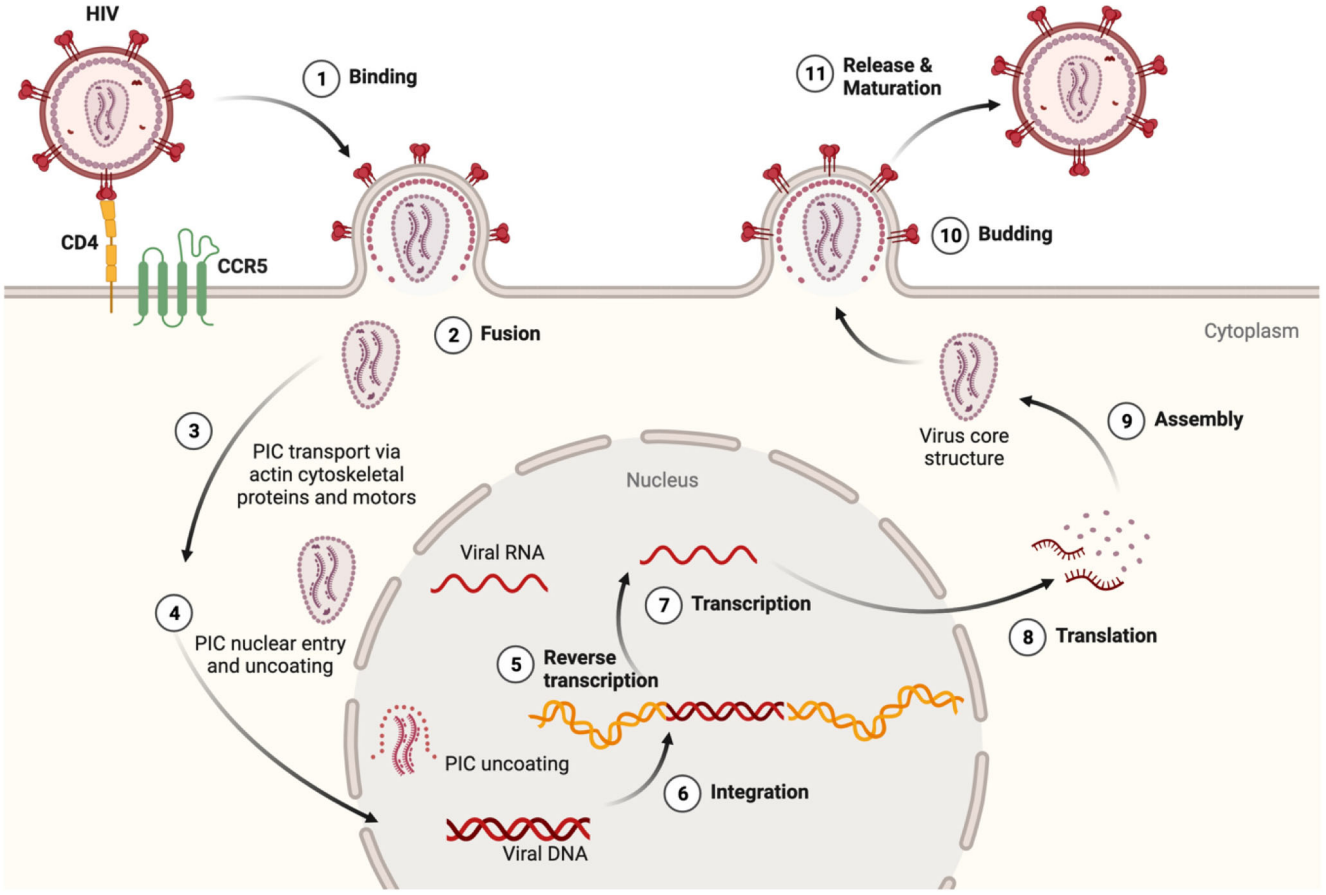
HIV lifecycle and relevance to inflammasome activation. (1) At target cell plasma membrane domains, HIV envelope protein gp120 trimer (red) binds to the CD4 receptor (yellow). Conformational alterations expose binding surfaces for coreceptor CCR5 on the Env trimer (green). (2) Fusion of Env with the cell plasma membrane is followed by uncoating and release of the preintegration complex (PIC) which contains a few molecules of reverse transcriptase, integrase and two copies of HIV RNA (vRNA) (29–31). Should the integrity of the PIC be compromised, viral RNA and proteins could be detected by innate immune sensors and thus initiate an inflammasome priming. (3) The actin cytoskeleton and specific microtubule motors transport the PIC to the nucleus (32). (4) The PIC can enter the nucleus in several ways including direct nuclear import and induced invaginations (24, 30). Degradation of the nucleoprotein coat would expose vRNA outside the nucleus, and provide another opportunity to activate innate antiviral responses. After reverse transcription (5), integration (6), transcription (7), and translation (8), viral proteins, vRNA, and (9) certain host proteins assembled at the inner plasma membrane surface. This mobilizes cytoskeletal proteins and molecular forces that facilitate budding (10), (11) release and maturation of new viral particles (32). Macrophages and microglia unlike T-cells are much more resistant to the cytopathic effects of HIV replication and therefore undergo innate immune activation in a sustained fashion. The figure was created with BioRender.

HIV encodes nine genes that co-opt intrinsic immune cell pathways normally used for growth, metabolism and homeostasis (24, 25). Innate immune signaling is an early detection system meant to thwart pathogen replication by activating the release of inflammatory molecules that, in turn, prime adaptive immunity (26–28). HIV-1 binds to CD4 and chemokine receptors, in a process that initiates fusion of the viral and plasma membranes (**Figure 1**). Neurons express chemokine receptors that support neuronal development and maturation, but not CD4, and therefore, do not allow HIV entry (33). Viral fusion is followed by the release and trafficking of the preintegration complex (PIC) to the nucleus (**Figure 1**). The PIC uncoating process within the nucleus was first shown for primary human macrophages years ago (29, 31), but only recently confirmed for T-cells (34). This mechanistic detail has important implications for understanding whether HIV can delay detection by nucleic acid sensors that activate Toll-like receptor (TLR) signaling (24, 30). Importantly, in MMs, virus is packaged in vesicular bodies and buds from the plasma membrane in contrast to the cytopathic release of viral particles from T-cells (35, 36).

Microglia not only protect the brain from pathogens and injury, but also serve critical roles in maintaining neuronal viability, proper synaptodendritic function and integrity in development and over the lifespan (20, 21, 37–39). Understanding the mechanisms by which HIV-1 affects microglial innate immune function is key to addressing the brain as a source of pathologic neuroinflammation correlated with neurological and psychiatric comorbidities (18, 23, 40, 41). Below, we discuss what is known about HIV activation of the inflammasome, particularly as it relates to microglia and neurons and the expression of specific pro-inflammatory cytokines that remain elevated in people with NeuroHIV. We then discuss another innate sensor, secreted phosphoprotein-1 (or osteopontin, *SPP1*/OPN), and its intersection with the mammalian target of rapamycin pathway (mTOR) and potentially the inflammasome to provide a unifying view of putative mechanistic connections and cell-type dependent crosstalk between the pathways.

## HIV activation of inflammasome signaling in the CNS

As the exploration of inflammasome function has progressed, NLRP3 is implicated in a variety of neurodegenerative diseases, including NeuroHIV (42–48). The inflammasome is a multiprotein complex involved in the immune and inflammatory response. Different inflammasomes types exist in the nucleotide-binding oligomerization domain, Leucine-rich-containing proteins (NLR) family (49). However, all inflammasomes contain key components including: NALP/NLR protein, PYCARD/ASC (Apoptosis-associated speck-like protein containing a CARD), and an enzyme responsible for pro-inflammatory cytokine activation (50–52). The NLRP3 inflammasome complex interacts with caspase-1 to activate IL-1β and IL-18 (53, 54). Both are pro-inflammatory cytokines that play various roles throughout the body. In microbial infections, the increase in IL-1β secretion is responsible for recruiting innate immune cells. In neurodegenerative diseases, IL-1β levels increase in response to microglial activation and neuronal injury (55, 56). IL-18 induces IFN production in T-cells and natural killer cells, promotes the production of other cytokines, and is suggested to exacerbate demyelination and cellular infiltration (44, 57).

**Figure 2:**
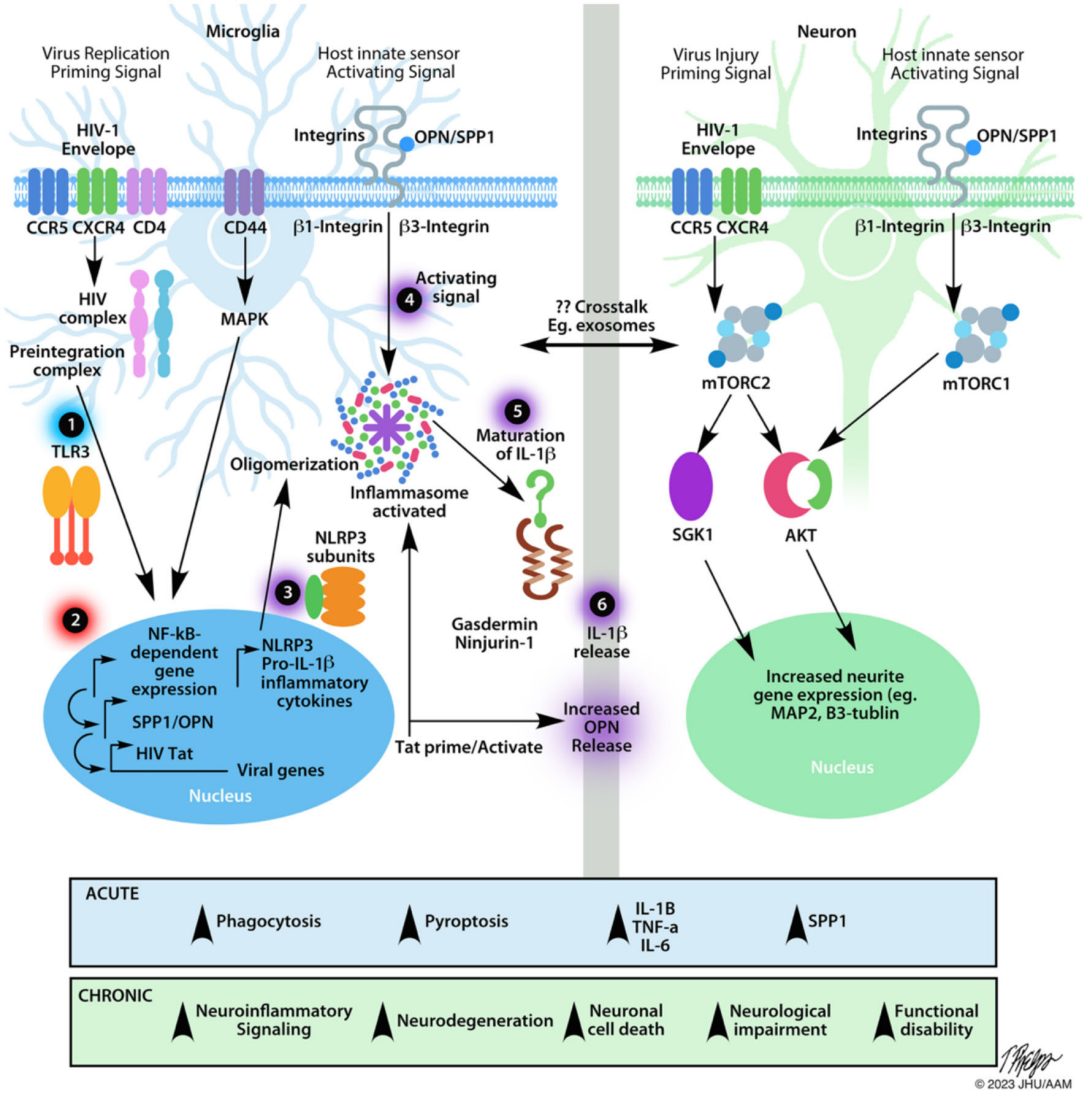
Schematic of the effects of HIV infection on microglia and neurons. The NLRP3 inflammasome is a multi-protein complex implicated in many neurodegenerative diseases including HAND. After HIV crosses the blood-brain barrier, it can bind to CD4+ cells, such as microglia, initiating the fusion of the virus to the plasma membrane, ultimately allowing HIV to enter the cell. After infiltrating the cell, many different aspects can affect the transcriptional activity of microglia via the NF-*κβ* pathway. Microglia. Step 1 indicates the first step required for inflammasome assembly: the priming step. Many stimuli can prime the NLRP3 inflammasome, including dsRNA for endosome-bound TLR3 (58, 59, 61, 62). Priming of the inflammasome leads to the localization of NF-*κβ* into the nucleus, indicated by Step 2. Along with host gene transcription, proteins like HIV TAT can be transcribed, which can act to prime/activate the inflammasome. Step 3 indicates the availability of the NLRP3 subunits necessary for the inflammasome to be oligomerized such as the NLRP3 protein, apoptosis-associated speck-like protein (ASC), and pro-caspase-1 (62). Step 4 indicates the activating step in NLRP3 inflammasome activation. Various stimuli, such as extracellular TAT protein, can trigger the activating signal. After receiving an activating signal, the NLRP3 inflammasome can begin its oligomerization and become functional. Pro-interleukin enzymes are recruited to be cleaved into their mature forms. For the NLRP3 inflammasome, IL-1B and IL-18 are cleaved by Caspase-1 and released, as shown by Steps 5, 6. The release of NLRP3-associated pro-inflammatory cytokines occurs via pores formed in the cell membrane. Caspase-1 will also cleave gasdermin D, leading to pyroptosis (62). The release of cytokines and viral proteins can then exacerbate local inflammation, leading to the recruitment of more immune cells and can affect other cell types, such as neurons. Neurons. Considering that HIV is unable to infect neurons directly, there are many examples of HIV-induced neuronal damage. One major contributor is the HIV-1 gp120 (Env). This protein can bind to CXCR4 and CCR5 receptors, expressed on neurons. HIV-1 Env has been shown to damage synaptic connections in cortical neurons when bound to CXCR4 via mTORC2 (63). When neurons were co-treated with HIV-1 Env and OPN/SPP1. Neurons showed signs of activated mTORC1/mTORC2 pathways, suggesting a regulatory feedback loop. Along with the required β1 and β3 integrin receptors, which OPN/SPP1 binds to, OPN/SPP1 acts as a neuroprotective modulator that promotes neurite growth in cortical neurons (β1 integrin) and regulates post-synaptic dendritic spine density in hippocampal neurons (β3 integrin) through mTORC1 (63, 64). Despite the protective effects of OPN/SPP1, over time, HIV-infected individuals present with neuronal degradation. Crosstalk. HIV-infected microglia have increased levels of NLRP3 activity, leading to pyroptosis and the release of highly pro-inflammatory cytokines. Given the role of IL-1β in inflammation, it is important to consider the various impacts it can have on the local microenvironment. Surrounding cells will respond to the inflammatory signal, such as upregulating SPP1/OPN. Regardless of the intent to reduce neuroinflammation, we see that HIV-infected individuals continue having low levels of chronic inflammation while on antiretroviral treatment. When looking at the acute effects, there is an increase in microglial phagocytosis, pyroptosis, pro-inflammatory cytokines, and OPN/SPP1 secretion. Chronically, we begin to see prolonged neuroinflammatory signaling, neurodegeneration, neuronal cell death, neuronal impairment, and functional disability, indicating the urgency to understand better the mechanisms of disease progression, cellular interactions, and regulation of neuroinflammatory pathways in HIV infection.

NLRP3 inflammasome assembly needs two signals: a priming and an activating signal (58–60). Of the many ways to prime the inflammasome, the most studied route is through NFkappaB-dependent signals (**Figure 2**). Many ligands can prime the NLRP3 inflammasome, including lipopolysaccharide (LPS) and TLR inducers like dsRNA (59–61). During reverse transcription, dsRNA can be detected by intracellular, endosome-bound TLR3 (**Figure 2**) (32, 65). TLR3 ligand binding activates ERK 1/2, MAPK, and NFkappaB-pathways, promoting gene transcription (62). Interestingly, the HIV transactivator of transcription (Tat) protein alone can prime and activate the inflammasome complex (**Figure 2**) (66). Various ligands such as, ATP, nigericin, aggregated proteins, reactive oxygen species (ROS), and HIV viral proteins activate the NLRP3 inflammasome (46, 49, 58, 61, 66–69). These signals allow for the recruitment of additional proteins like NLRP3, ASC, and caspase-1 that are necessary for oligomerization and subsequent cleavage and maturation of cytokines (62). Caspase-1 also cleaves gasdermin D, leading to cell membrane pore formation, and a type of pro-inflammatory cell death known as pyroptosis (**Figure 2**) (62).

The NLRP3 inflammasome is robustly expressed in microglia (42, 59). However, whether the same is true for neurons is less well known. Interestingly, neurons undergoing pyroptosis have been documented (70–72). This is important since pyroptosis is strongly associated with NLRP3 inflammasome activation (73–77). The NLRP1 and AIM2 inflammasome complexes of cortical neurons have been the most investigated (70–72). Recently, studies reported that dopaminergic neurons express NLRP3 throughout the progression of Parkinson’s disease (45, 47). However, activation of NLRP3 in microglia contributes to demyelination through IL-1β and IL-18 secretion (44). HIV-positive individuals have increased caspase-1, IL-1β, and IL-18 levels, suggesting NLRP3 inflammasome activation systemically and in the CNS (78–80). Given the association between neurologic disorders, neuroinflammation, and the activation of the NLRP3 inflammasome in microglia and neurons, the potential for crosstalk between these cells is expected.

## HIV induced inflammasome activation and mTOR signaling in NeuroHIV

There is renewed interest in mTOR signaling in HIV infection as new roles for this pathway have emerged. Early studies implicated a role for mTORC signaling in promoting virus replication (81–83). Most recently, mTORC-regulated mechanisms in HIV escape from latency in T-cells (84), autophagy (85), apoptosis (86), and the homing of intestinal CCR6+CD4+ T-cells (87) have been reported. Interestingly, in efforts to identify new candidate genes involved in latent HIV infection, a role for pro-inflammatory cytokines and signaling pathways regulated by secreted phosphoprotein-1/osteopontin (*SPP1/*OPN) were discovered (88). The mTOR pathway is composed of two structurally distinct, multi-subunit protein complexes, mTORC1 and mTORC2 that receive signals about a cell’s metabolic status to fine tune growth and repair processes through activation of relevant transcriptional programs (89, 90). HIV-positive individuals have dysregulated autophagy, indicating upregulated levels of mTOR activity (91). Increases in mTOR activity are associated with reactive microglia, neuronal damage, neurodegeneration, and memory deficits, all characteristics of NeuroHIV (63, 92). Although scarcely investigated, evidence of a regulatory relationships between mTOR and NLRP3 in immune cells and neurons have been reported. Studies have shown that downregulating mTOR activity reduces NLRP3 activation (93–96). With reduced mTOR activity, autophagy removes detrimental pro-inflammatory stimuli, including ROS. Indeed, ROS activates the NLRP3 inflammasome and has been associated with NeuroHIV (97, 98). Another study found that inhibition of mTORC1 leads to decreased secreted IL-1β, indicating post-transcriptional effects on NLRP3 activation (94). A similar regulatory relationship was observed with *in vitro* and *in vivo* NLRP3 knock-out studies in which mTOR activity decreased (93, 99). In macrophages an interaction between NLRP3 and mTOR was found, indicating a direct protein-protein interaction and communication between both pathways (93). Lastly, IL-1β can activate mTOR in T-cells and in hippocampal neurons further illustrating NLRP3 cell-specific- and cell-to-cell communication pathways and functional outcomes like neuroinflammation (100–102). The emerging relationships between NLRP3, mTOR, and HIV infection becomes more interesting when considering the function of additional innate immune sensors like *SPP1/*OPN.

## Innate signaling pathways collide: SPP1/OPN and mTOR activation in NeuroHIV

The term neuroinflammation, as it is currently understood, broadly signifies a mix of innate and adaptive responses of resident brain- and circulating immune cells that, if left unregulated, can have damaging short- and long-term consequences (103). In this regard, chronic expression of proinflammatory molecules leads to over activation of the immune system and accumulation of damage and disability with time. Secreted phosphoprotein-1 (*SPP1/*OPN), by virtue of its modular domain structure, is a multifunctional phosphoprotein implicated in several neurodegenerative diseases (104–110). The expression of *SPP1/*OPN is markedly elevated in the CNS of humans and non-human primate models of HIV infection (109, 110). However, more recent findings with humanized mice and positron emission tomography neuroimaging demonstrate that *SPP1*/OPN expression is required to downregulate the microglial inflammatory response (111). How exactly *SPP1*/OPN modulates the HIV-induced inflammatory response in the brain is not yet understood. However, in cultured primary human macrophages, HIV replication and NF-*κβ* activity is increased in the presence of *SPP1/*OPN (110). The degree of neuroinflammation correlated with the extent of HIV replication only in humanized mice expressing *SPP1/*OPN (111). Neurons cannot be infected with HIV due to their lack of the CD4 receptor, however the presence of certain chemokine coreceptors like CCR5 or CXCR4 makes them vulnerable to excitotoxicity, degeneration and death after binding interactions with HIV Gp120 (33, 112). However, treatment of neurons with recombinant OPN protects hippocampal post-synapses from synaptodendritic injury, and the structural integrity of cortical axons and dendrites via mTORC1/mTORC2 activation (**Figure 2**) (63, 64). Therefore, in NeuroHIV, increased expression of *SPP1*/OPN is largely neuroprotective.

## The intersection of SPP1/OPN, mTOR and inflammasome signaling in neurodegenerative disorders

We first hypothesized that the overexpression of *SPP1/*OPN in individuals with NeuroHIV was harmful, but as discussed above the findings thus far point to a neuroprotective function. While there is increasing evidence of linkages between neurodegeneration and cellular repair processes involved in resolving neuronal injury and neuroinflammation, significant gaps in our understanding of the molecular mechanisms remain. *SPP1/*OPN was identified as a highly-expressed transcript that clustered with a collection of genes termed “disease-associated microglia (DAM) (113–115). Recent studies by Rentsendorj et al., and Qiu et al., beautifully demonstrate using the ADtg and 5XFAD mouse models for AD, respectively roles for specific populations of *SPP1+/-* expressing monocytes, resident microglia and/or macrophages in the phagocytosis of amyloid and speculate about a role for inflammasome signaling (116, 117). In contrast, in a slow-progressing model of AD (App^NL-F^ knock-in reporter mice), *SPP1*+ macrophages and microglia associated with brain blood vessels and those located in the hippocampus were responsible for pathologic microglia-synapse destruction (118, 119). In another example of neuroprotection, regulatory T-cells localized in the brain several weeks after stroke express *SPP1/*OPN and, through a microglial-β1-integrin-dependent manner, foster repair of white matter axonal damage (120). In a model of glaucoma, a protective role for SPP1/OPN was found (117). Interestingly, in an ischemia model, intranasal delivery of a *SPP1/*OPN peptide suppressed microglial activation and the release of pro-inflammatory cytokines IL-1β and IL-6, an indication of reduced NLRP3 activity (121). To further support this idea, Zhang et al. demonstrated that *SPP1/*OPN negatively regulates the NLRP3 inflammasome in ischemic infarction (122). Lastly, in a MS model, NLRP3 knockout, as well as one of its components ASC, reduced mRNA SPP1/OPN expression in splenic CD4+ T cells (123). Whether this same relationship exists in the CNS is unknown, though it is possible that NLRP3 priming lead to NF-kappaB transcription of *SPP1/OPN*. Given its neuroprotective function, a negative feedback loop may be in place to prevent chronic inflammation via continuous NLRP3 activation. Importantly, as more details on the molecular mechanisms of *SPP1/*OPN function continue to emerge, the information will help provide a more complete understanding of the correlative findings of clinical studies (124) and toward the design of possible efficacious therapeutic interventions.

Over the last several years, understanding of the direct role of glycolytic metabolism on effector immune cell functions has greatly increased (125–128). As such, there are opportunities for pathogens to alter and/or harness signaling dynamics that feed directly into the mTOR pathway (129–132). Tissue macrophages and microglia assume a variety of activation states in response to local cues, and downstream stimulation of mTOR signaling is implicated in their M2- (anti-inflammatory) or M1-polarization (proinflammatory), respectively (133). Interestingly, inhibition of inflammasome activation is protective against disease progression in a mouse model of multiple sclerosis. In this regard, rapamycin, an immunosuppressive agent, was shown to block antigen presentation by dendritic cells and inflammatory signaling by microglia (133, 134).

The homeostatic balance of the immune system is maintained through direct and indirect interactions and with soluble factors acting locally and over long distances (refs). HIV infection disrupts and hijacks the important cell-to-cell communication network. The virus infects T-cells and MMs robustly and astrocytes in a limited fashion (35), and cells located nearby initiate a signaling cascade that amplifies locally, and recruits additional immune cells from a distance. This idea of cellular crosstalk was investigated by Wang and Gabuzda, who saw that direct contact between neurons and microglia was not necessary for neuronal damage (135). The same study also found that activated astrocytes promoted HIV replication in microglia. In this regard, as discussed above, mTOR signaling in cultured cortical neurons preserves structural integrity, however increased mTOR activity can also be detrimental to cells of the brain (133, 134, 136). Cortical neurons, as well as infected microglia may, in turn, be upregulate and secrete OPN/SPP1 to reduce the inflammatory response by inactivating the NLRP3 inflammasome in microglia, and promoting neuronal survival through mTOR activity. Decreased mTOR activity in astrocytes is primarily beneficial, but negatively affects their ability to differentiate (133, 134, 136). In oligodendrocytes, decreased mTOR activity impairs their differentiation and myelination functions (133, 134, 136). The release of damage signals and proinflammatory molecules from impaired glial cells, activates immune cells and neurons thus amplifying a neuroinflammatory response. An example being the rapid release of IL-1β and IL-18 from microglial pyroptosis (**Figure 2**). We emphasize the importance of considering that homeostasis in chronic low-level HIV infection is tightly regulated via crosstalk between different cells through secreted pro- and anti-inflammatory cytokines/chemokines. The delicate balance, or lack thereof, of a cellular local environment, can act to exacerbate or ameliorate neuroinflammation. Indeed, HIV utilizes these delicate communication pathways to promote an optimal environment for replication.

Given that microglia have receptors for OPN, it’s possible that signaling by cortical OPN/SPP1 via mTOR acts on microglia to reduce the inflammatory response and increase transcriptional programs involved in preserving neuronal function. Given their opposing, yet collaborative, roles in inflammation, it is important to investigate the relationship between *SPP1/*OPN, mTOR, and NLRP3 in HIV-induced neuroinflammation and NeuroHIV. In this regard, more research is needed to get a better understanding of the molecular and cellular mechanisms that take place in chronic HIV infection. Doing so would allow us to understand better how HIV manipulates the host’s protective measures, allowing for better treatments aimed to improve the host response to latent HIV infection, guiding us toward a solution to eliminate HIV-associated neuroinflammation and cognitive deficits.

## Discussion

There is a greater appreciation that during development and adulthood, dynamic homeostatic regulation of the brain’s neural network is intertwined with and dependent on crosstalk and connectivity with glial. Disruption of the integrity of the brain, as seen in viral infection, leads to activation of what are meant to be protective responses, resulting in a neuroinflammatory response involving resident brain cells and immune sentinels that conduct tissue-level surveillance. As reviewed herein, innate immune signaling, including mTOR, SPP1/OPN, and NLRP3 inflammasome activation, is initiated to monitor and/or alter cell metabolic state, stimulate repair, migration, and other immune effector processes. Given that several myeloid and glial cells and cofactors can contribute and stimulate autocrine and paracrine feedback and feed-forward looping, how are the outputs integrated to restore homeostatic levels of regulation and surveillance? Deeper insight into the physiological, cellular, and molecular mechanisms will help to advance the development of effective interventions to help those suffering from neurological and neuropsychiatric comorbidities related to chronic over-activated innate immune responses in the central nervous system.

## Author contributions

AB: Conceptualization, Funding acquisition, Writing – original draft, Writing – review & editing. CA: Conceptualization, Writing – original draft, Writing – review & editing.
